# Rare case of fecaloma requiring endoscopic intervention in a 24-year-old female

**DOI:** 10.1093/jscr/rjae438

**Published:** 2024-07-08

**Authors:** Zamaan Hooda, Sabrina Clare Higgins, Dakota Pastore, Gregory Crisafulli, Haley Canoles, Mina Alkomos, Toghrul Talishinskiy, Kamal Amer, Abraham El-Sedfy

**Affiliations:** Department of Surgery, St. Joseph’s University Medical Center, 703 Main Street, Paterson, NJ 07503, United States; Department of Surgery, St. Joseph’s University Medical Center, 703 Main Street, Paterson, NJ 07503, United States; Department of Surgery, St. Joseph’s University Medical Center, 703 Main Street, Paterson, NJ 07503, United States; Department of Surgery, St. Joseph’s University Medical Center, 703 Main Street, Paterson, NJ 07503, United States; Department of Surgery, St. Joseph’s University Medical Center, 703 Main Street, Paterson, NJ 07503, United States; Department of Surgery, St. Joseph’s University Medical Center, 703 Main Street, Paterson, NJ 07503, United States; Department of Surgery, St. Joseph’s University Medical Center, 703 Main Street, Paterson, NJ 07503, United States; Department of Surgery, St. Joseph’s University Medical Center, 703 Main Street, Paterson, NJ 07503, United States; Department of Surgery, St. Joseph’s University Medical Center, 703 Main Street, Paterson, NJ 07503, United States

**Keywords:** fecaloma, sigmoidoscopy, large bowel obstruction, constipation

## Abstract

Fecalomas are a rare potential etiology for constipation experienced in children and the elderly. Large bowel obstructions due to fecalomas are preferably treated conservatively with laxatives and bowel rest. However, in the setting of severe corporostasis, more invasive procedures are required to prevent bowel ischemia and perforation. This case report describes a patient who presented to the emergency department with symptoms of large bowel obstruction and constipation, and she was found to have a fecaloma. Conservative interventions, including bowel rest and the administration of laxatives failed, prompting the need for more invasive therapies. During her admission, multiple flexible sigmoidoscopies were required to alleviate the obstruction. Ultimately, this case demonstrates an encounter of a patient with a sigmoid fecaloma from an unlikely demographic with few risk factors that required endoscopic intervention for treatment.

## Introduction

A fecaloma is a collection of dry, impacted stool that has hardened, which forms in the context of coprostasis. Ultimately, it develops into an intraluminal mass most commonly found in the sigmoid colon and rectum. The prevalence of fecalomas occurs in a bimodal distribution, affecting children and the elderly [1, 2]. Patients at risk for developing fecaloma include those who are frail, immobile, or have a history of chronic constipation. Fecalomas also tend to occur more frequently in patients with neuropsychiatric diagnoses as well as those taking chronic opioid analgesics [1]. Additionally, acquired, congenital or inflammatory conditions, such as Hirschsprung’s disease and irritable bowel syndrome, have also been associated with fecalomas [2].

Young patients seldom present with fecal impaction. These rare cases are typically associated with concurrent chronic constipation or irritable bowel syndrome-like symptoms [2, 3]. Uncommonly, cases of foreign body ingestion may be implicated in large bowel obstruction in adolescents and young adults [3, 4]. If left untreated, fecalomas can enlarge, which can cause mass-effect complications, such as bowel obstruction and stercoral perforation [5]. Rarely, fecalomas may lead to megacolon and abdominal compartment syndrome. For these reasons, prompt evaluation and therapeutic intervention are recommended [6–8]. In this report, we highlight a rare case of a 24-year-old female presenting with symptoms of large bowel obstruction due to fecaloma.

## Case report

A 24-year-old female patient presented to the emergency department with complaints of abdominal pain and constipation for three days. The patient had a medical history of bipolar disorder, which was well controlled with daily Lamotrigine. She reported taking a stool softener with limited relief. She was initially treated for her symptoms and discharged home. She returned to the emergency department 5 days later with complaints of continued symptoms.

**Figure 1:**
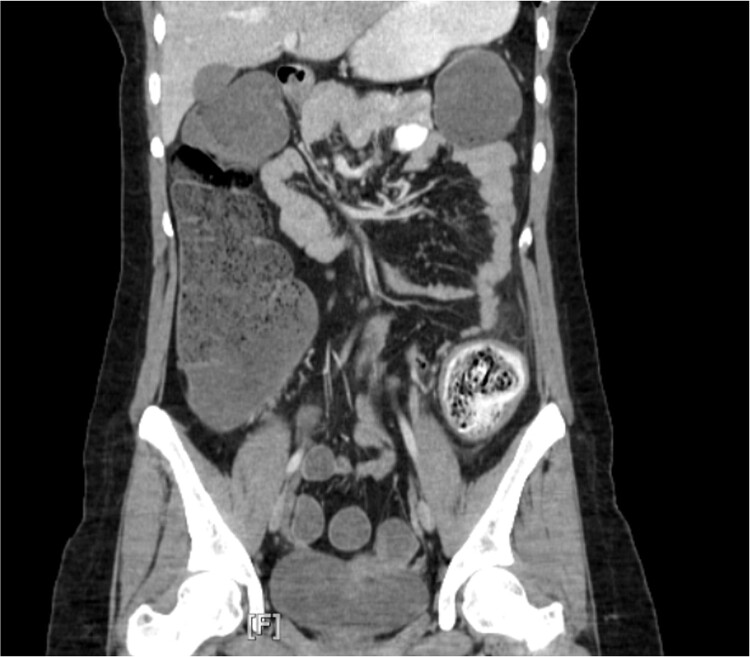
Computed tomography imaging revealed a mechanical bowel obstruction at the distal descending colon secondary to impacted fecal matter with noted colonic wall thickening and edema.

Repeat CT abdomen and pelvis (Fig. 1) found a mechanical large bowel obstruction secondary to distal impacted fecal matter in the descending colon along with segmental wall thickening with concern for fecaloma. Upon further questioning, the patient denied any ingestion of foreign objects or any previous history of irritable bowel syndrome. She mentioned adequate daily fluid intake and a moderate-fiber diet. She also mentioned never having any issues of constipation in the past, and denied any personal or family history of ulcerative colitis or Crohn’s disease. Due to the longevity of symptoms and CT scan findings, the patient was admitted with consultation to colorectal surgery and gastroenterology. Additionally, a bowel regimen with senna glycoside and docusate sodium was administered.

**Figure 2:**
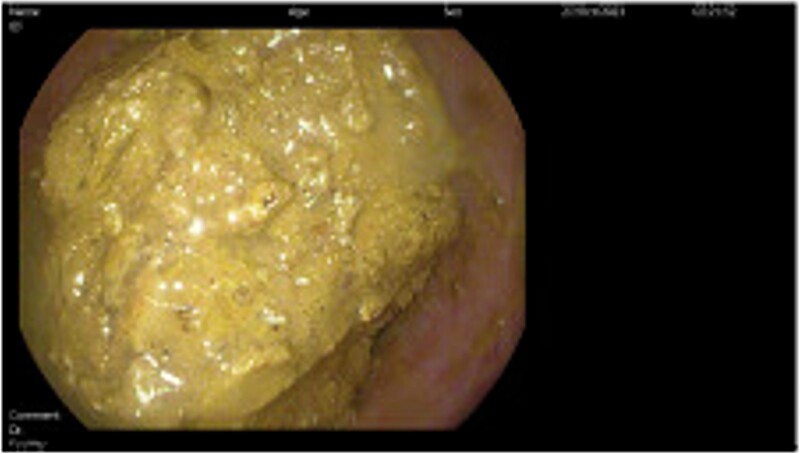
Direct visualization of the fecaloma causing colonic obstruction was obtained during the endoscopic intervention performed during hospital admission.

Subsequently, the patient underwent a series of three sigmoidoscopies on Day 2, 3, and 5 of her hospital stay (Fig. 2) after receiving polyethylene glycol prior to each intervention. During the first procedure, attempts at disimpaction of the fecaloma were made. Techniques used included saline lavage along with manual disimpaction with forceps and snares. Despite these efforts, disimpaction of the fecaloma was only minimally successful, leaving a significant amount of retained stool in the descending colon. The procedure was aborted, and the bowel regimen was continued along with the administration of a fleet enema; these measures brought only minimal relief. The following day, another sigmoidoscopy was performed, revealing a 35–40 cm fecaloma almost completely obstructing the bowel lumen with surrounding mild inflammation and ulceration. Attempts were made to dislodge the fecaloma using a net grasper, biopsy and snare, infusion of hydrogen peroxide diluted with saline, and an injection with mineral oil and an endoscopic retrograde cholangiopancreatography balloon. However, this attempt was unsuccessful, and a decision was made to repeat the sigmoidoscopy after giving the patient polyethylene glycol and fleet enemas for 2 days. Following this, the patient was able to pass more flatus and had a small watery bowel movement.

During the final sigmoidoscopy, successful snaring was achieved, allowing for a small mobile piece of the fecaloma to bypass the transverse colon. Additional evidence of edema and hyperemia in the descending colon as well as erosions in the sigmoid colon were appreciated without evidence of perforation or ischemia. No surgical intervention was warranted due to minimal concerns about perforation. The patient was discharged on hospital Day 6 with instructions to follow up with gastroenterology for a completion colonoscopy to ensure no evidence of inflammatory bowel disease.

## Discussion

This case describes a rare presentation of fecaloma in a young female patient that does not fit the typical patient population that experiences chronic constipation, less so fecalomas. Despite having bipolar disorder, the patient was functional and independent. Furthermore, the medication the patient took for bipolar disorder, Lamotrigine, is not commonly associated with constipation, making it an unlikely culprit for the cause of the fecaloma formation [9]. She also did not have any medical history of irritable bowel syndrome, chronic constipation, or any spinal cord injuries. Additional questioning of the patient’s psychosocial and dietary habits revealed no major risk factors to explain the fecaloma formation.

Outside of typical patients, current reports also describe congenital abnormalities creating fecal impactions and fecalomas in children. However, there is a paucity of literature regarding similar presentations in young adults that do not have the usual comorbidities leading to these pathologies. Recently, there has been an increase in the incidence of fecalomas occurring in young adults in this specific patient population with only singular risk factors, such as constipation [10–13]. This raises the question of whether guidelines should recommend a lower threshold for investigating fecalomas and also calls for further investigation into adequate preventative measures that can be taken.

Although fecal impaction and fecalomas usually have a benign disease course, they can rarely lead to stercoral perforation and peritonitis, which is a surgical emergency that puts patients at risk of severe morbidity and mortality [2–6]. Currently, conservative management, including bowel rest, fluid resuscitation, fiber supplementation, laxatives, and manual digital evacuation, are first-line treatments for fecalomas [8–10]. When these options fail, endoscopic evacuation, and possible surgical interventions are pursued [11, 14]. Despite success, other techniques for endoscopic treatment of fecalomas are being proposed, including the injection of Coca-Cola. The use of Coca-Cola has been described as a successful method to dissolve phytobezoars, which have a similar composition to fecalomas [15]. Although the data regarding this method is scarce, the current literature shows promise, and further investigation is warranted to determine its efficacy.

## Conflict of interest statement

None declared.

## Funding

No external funding was necessary or given for this manuscript.
